# Prevalence of blood borne viruses in IVF: an audit of a fertility Centre

**DOI:** 10.5935/1518-0557.20160030

**Published:** 2016

**Authors:** Michael B Yakass, Bryan J Woodward, Mary A Otoo, Edem K Hiadzi

**Affiliations:** 1 Assisted Conception Unit, Lister Hospital & Fertility Centre, Accra, Ghana; 2 IVF Consultancy Services, Leicester, UK

**Keywords:** Hepatitis B, Hepatitis C, HIV, sperm quality, IVF

## Abstract

**Objective:**

The rate of infertility continues to be on the increase in the developing world. Similarly, the rates of blood-borne viral infections (BBVs) such as Human Immunodeficiency Virus (HIV), Hepatitis B virus (HBV) and Hepatitis C virus (HCV) are also on this rise. In 2014, the World Health Organization (WHO) quoted prevalences of 1.5% (HIV), 15% (HBV) 1.3 - 8.4% (HCV) in the Ghanaian general population. It has been reported that BBVs can adversely affect male fertility, specifically sperm count and progressive motility. The aim of this study was to evaluate the prevalence of BBVs in people with infertility attending an IVF clinic and whether or not BBVs impacted on sperm parameters.

**Methods:**

A retrospective cohort study at a private fertility center in Accra, Ghana. We had 229 recruited couples assayed for HBV, HCV and HIV. Sperm parameters of the male partners were also assessed. The analysis performed included student t-test and Fisher's exact test.

**Results:**

We found prevalence rates of 1.7% (HIV), 7.9% (HBV) and 0.4% (HCV), which is similar to what has already been reported in the Ghanaian community. There was no significant difference between BBV positive and negative subjects for sperm count (13.6 million/ml vs. 17.7 million/ml, *P* = 0.0599), percentage of progressive motility (26% vs. 30%, *P* = 0.2129), percentage of normal forms (3% vs. 3%, *P* = 0.0617) and clinical pregnancy rates per embryo transfer (36.1% vs 34.9%, *P* = 0.5) between BBV positive and BBV negative subjects, respectively.

**Conclusion:**

There is a similar prevalence of BBVs in sub-fertile couples and the general Ghanaian population. However, no detrimental effect has been reported for sperm parameters on grounds of BBV infectivity of the male partner.

## INTRODUCTION

Worldwide more than 70 million, or 10% (Boivin *et al*., 2007) of couples, suffer from infertility, with a high proportion living in developing countries (Ombelet *et al*., 2008). The inability to have children can have negative emotional, psychological and social impacts on the lives of these people (Ombelet *et al*., 2008; Rouchou, 2013). In general, secondary infertility has been shown to dominate over primary infertility in most developing countries, and this is chiefly a result of improperly treated sexually transmitted infections (STIs), resulting in pelvic inflammatory disease (PID) (Larsen *et al*., 2006; Elussein *et al*., 2008).

It is estimated that 40 million people worldwide are living with HIV/AIDS and approximately two thirds of those live in sub-Saharan Africa (WHO, 2015). The World Health Organization (WHO) quotes a prevalence of 1.1 - 2.0% in the Ghanaian general population (WHO, 2015). The national sero-prevalence of HIV was estimated to be 1.5% in 2013 by the national AIDS control program in Ghana (Ghana Health Service, 2008). 

HIV-infected people were initially discouraged from starting a family (Savasi *et al*., 2013). However, with the introduction of antiretroviral therapies, life expectancies and the quality of life has dramatically improved and many infected people are now thinking of starting a family, usually via assisted reproductive technology (ART) (Savasi *et al*., 2013).

Several studies have documented reduced fecundity in HIV-infected individuals compared to uninfected people (Glynn *et al*., 2000, Brocklehurst & French, 1998). On the other hand, marital instability and polygamy, secondary to infertility, may in turn increase the spread of HIV-1 infection (Ombelet *et al*., 2008).

The prevalence of chronic Hepatitis B virus (HBV) infection varies widely according to geographical area. Sub-Saharan Africa is endemic in HBV with an estimated 5-25% being chronic carriers (Candotti *et al*., 2007). HBV prevalence in Ghana has been estimated to be around 15% (Ghana Health Service, 2008).

The prevalence of the Hepatitis C Virus (HCV) has been reported to be >1% in southern African countries, 1.7 - 27.5% in central Africa and 1.4 - 7% in West and East Africa (Candotti *et al*., 2003). The estimated serum-prevalence of HCV is 1.3-8.4% among blood donors in Ghana (Ampofo *et al*., 2002).

In general, BBV infections have been shown to contribute to male infertility either by direct toxic effects on cells in the male reproductive tract, and/or indirectly by causing a local inflammatory or immunological reaction (Zhou *et al*., 2011). HBV infection has been reported to increase chromosomal instability in sperm and impair overall sperm quality (Huang *et al*., 2003, Huang *et al*., 2002). Furthermore, HBV has been linked to decreased sperm motility (Lorusso *et al*., 2010). However, other studies have reported no significant difference in sperm quality between HBV -serum-positive and -negative men (Zhou *et al*., 2011). Reduced implantation and pregnancy rates have also been shown following IVF treatment for people with HBV compared to age-matched controls 

(Pirwany *et al*., 2004).

The handling of potentially BBV-infected body fluids, gametes or embryos is a risk to healthcare professionals, such as physicians, nurses and embryologists. In addition, uninfected couples being treated at the same time may be at risk of nosocomial contamination (Lesourd *et al*., 2000). This is why strict adherence to the testing of all people seeking ART for HIV, HBV and HCV is mandatory (The Commission of the European Communities, 2006; Practice Committee of American Society for Reproductive & Practice Committee of Society for Assisted Reproductive, 2008). 

The aim of the current study was to calculate the BBV prevalence in people seeking ART at a private fertility clinic in Accra, Ghana. We also investigated if BBVs have any effect on sperm parameters.

## MATERIALS AND METHODS

Subjects were recruited between March 2013 and July 2015. Participating subjects signed consent forms to participate in the study, which was approved by the hospital's ethics and practice committee. 

Two hundred and twenty nine (229) heterosexual couples were recruited for this study, having complete viral screening results (HIV, HBV, HCV). Recipients of donor gametes (either sperm or oocyte) were included, provided the other gamete originated from a BBV-infected partner. Patients receiving frozen embryo transfers were excluded. 

For BBV analysis, 5ml blood was collected by venipucture into serum separator tubes and centrifuged at 500g for 5 minutes to separate the serum from cells. HBsAg, Anti-HIV 1 and 2 and HCV were determined from the serum with rapid diagnostics kits (Tellmefast, Biocan Diagnostics Inc, Canada). Quality control checks were performed daily before running assays.

The IVF stimulation protocol was as follows; down-regulation was achieved with 0.5 units of buserelin administered from Day 2 of the menstrual cycle till HCG administration. An ultrasound scan was performed between 14 - 21 days after starting buserelin injections to assess ovarian status and endometrial thickness. When down regulation was achieved, controlled ovarian stimulation (COS) was initiated alongside the buserelin administration. For the COS, 225 - 400 IU of recombinant FSH (Fostimon, IBSA, Switzerland) was administered daily for 7 - 10 days. An ultrasound scan was performed to assess follicular response between 5 - 7 days of COS and dosage adjusted accordingly when required. HCG (10,000 IU) (Choriomon, IBSA, Switzerland) was administered when the leading follicle was at least 18mm. Ultrasound-guided follicle aspiration was performed using a 17G Cook aspiration needle (Cook, Australia) 36 hours after the HCG injection. 

Semen samples produced on the day of the IVF/ICSI procedures were analyzed according the latest WHO laboratory manual for the examination and processing of human semen (Cooper *et al.*, 2010). 

The semen was prepared by the density gradient technique of sperm preparation. 1ml 40% gradient was gently over-layered onto 1ml 80% gradient (Global, IVF Online, Denmark) and warmed in the incubator set to 37°C for 30 minutes. 1ml of the semen was gently over-layered on the 40% gradient and centrifuged at 300g for 10 minutes. The supernatant was gently aspirated and discarded. About 0.3ml of the remaining pellet was aspirated and transferred into 3ml of AllGrad sperm washing solution (Global, IVF Online, Denmark) and centrifuged at 300g for 5 minutes. The supernatant was gently aspirated and discarded. A sperm count and motility assessment was then performed on the washed pellet using a sterile technique. This pellet was kept for use in the IVF procedure. 

Semen samples from BBV infected males were also processed using the density gradient centrifugation method with three gradient layers 90%, 70% and 40%, under a sterile technique. The removal of the supernatant at each step prior to transfer of the pellet helped minimize any viral transmission (Zamora *et al*., 2016).

Developing embryos from BBV-positive couples were cultured in separate gassed incubators (5% CO2, 6% O2 and 89% nitrogen, BOC, UK) from BBV-negative couples, to eliminate any risk of cross-contamination as per best practice (Magli *et al*., 2008).

A pregnancy test was performed on the serum of the female partners two weeks after the embryos were transferred.

Results were expressed as mean ± SEM or mean (range). The data was analysed using the Graph Pad Prism - version 5 (Graph Pad Software, San Diego California). Student t-test and Fisher's exact test were used to assess significance. Statistical significance was set at *P*<0.05

## RESULTS

Twenty-three (23) couples had repeat IVF treatments. One (1) female partner and four (4) male partners tested positive for HBsAg in both cycles. Two (2) male partners who tested negative at the first IVF cycle tested positive for HBsAg in the second, despite the advice and the availability of the hepatitis B vaccine at the Public Health Unit of the hospital. The time interval between both cycles was twelve (12) months for one male and 21 months for the other. Primary infertility was dominant over secondary infertility in our study population (Table 1). 

**Table 1:** Demographics of the general populationmigration pattern.

Parameters	BBV Positive	BBV Negative	*P* - value
Age (y)			
-Males	40 (34 - 51)	43 (32 - 59)	0.1097
-Females	36 (21 - 49)	38 (19 - 53)	0.0564
Duration of infertility (months)	72 (7.0 - 180.0)	84 (12.00 - 240.0)	0.0761
Primary Infertility %	76.2	23.8	
Secondary infertility %	85	15	
BMI (kg/m2)	26.92 ± 0.30	27.47 ± 1.80	0.5492

Data presented as Mean (Range) or Mean ± SEM

HBV prevalence was higher in the study population compared to their HIV and HCV infected counterparts. More men were significantly infected with the HBV than women (*P*=0.0027). The study did not find any significant difference in semen quality, i.e. sperm count, percentage progressive motility and percentage normal forms of BBV positive and BBV negative males. However, there was a trend for mean sperm count and percentage progressive motility to be higher in the BBV-negative males, although this was not significantly different from BBV-positive males (Table 2). 

**Table 2:** Prevalence of various blood borne viruses and their effects on semen quality.

**Parameters**		***P* - value**	
**Source of oocyte**			
Self	64.2%		
Donated	35.8%		
**HIV prevalence**			
Male	2.2%	0.7241^a^	
Female	1.3%		
Total prevalence	1.7%		
**Hepatitis B prevalence**			
Male	11.8%	0.0027^a^	
Female	3.9%		
Total prevalence	7.9%		
**Hepatitis C prevalence**			
Male	0.4%		
Female	0.4%		
Total prevalence	0.4%		
	**BBV Positive**	**BBV Negative**	***P* - value**
**Semen quality**			
Sperm count	13.6 ± 2.34	17.7 ± 0.78	0.0599
% motility	26 ± 2.4	30 ± 1.0	0.2129
% Normal forms	3 ± 0.3	3 ± 0.2	0.0617

Data presented as percentages (n/N), mean ±SEM.

a = Fisher’s exact test was performed.

Significance considered at P< 0.05

On account of the high prevalence of HBV, the effect of this virus on semen was assessed separately (Table 3). However, we found no significant difference in semen quality between HBV-infected males and those not infected. 

**Table 3:** Effects of HBV on semen quality.

	HBV Positive	HBV Negative	*P* - value
Sperm Concentration (million/ml)	13.5 ± 2.7	17.7 ± 0.8	0.0837
Progressive Motility (%)	25 ± 2.7	30 ± 1.0	0.1583
Normal forms (%)	3 ± 0.3	3 ± 0.2	0.0838

Data presented as mean ± SEM.

There was no statistical difference in semen quality when HIV infected males were compared to their HIV uninfected males (Table 4). All 5 males and the 3 females infected by the HIV were on the highly active anti-retroviral therapy (HAART).

**Table 4:** Effects of HIV on semen quality.

	HIV Positive	HIV Negative	*P* - value
Count	9.1 ± 3.8	17.0 ± 0.76	0.1383
% Motility	26.0 ± 6.4	29.0 ± 0.98	0.6264
% Normal Forms	2.5 ± 0.29	3.4 ± 0.15	0.4068

Data presented as mean ± SEM.

There was only 1 male infected with the HCV hence comparative analysis of semen quality between HCV positive and HCV negative males could not be performed due to small numbers.

**Figure 1:**
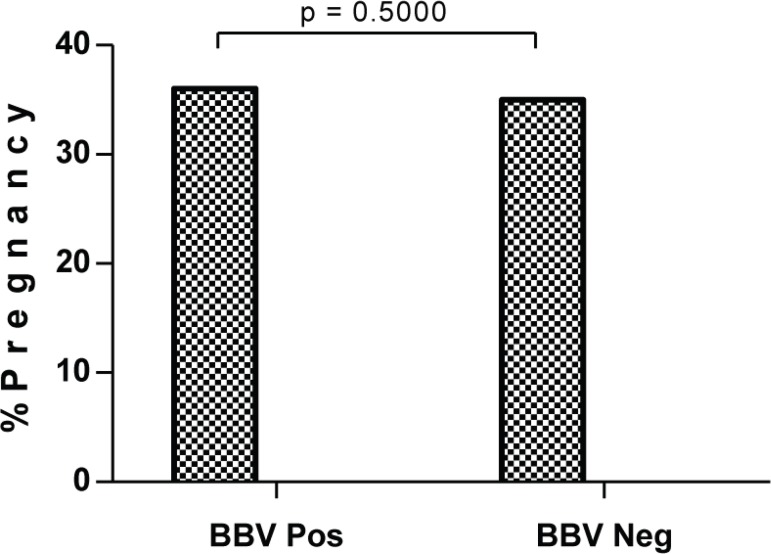
Pregnancy percentage differences by way of BBV infection.

We did not find any significant difference for clinical pregnancy rates between BBV-infected and uninfected couples (Figure 1). Interestingly, BBV-infected women had slightly higher pregnancy rates than those without BBV infection (36.1% vs 34.9%, *P* = 0.5000, 1-tailed).

## DISCUSSION

BBV (HIV, HBV and HCV) prevalence in our study population was similar to that found in other studies (Duda *et al*., 2005). Techniques, such as the density gradient sperm preparation, have been shown to significantly reduce the risk of transmission of viral infection from parent to offspring, especially if the male is infected (Zafer *et al*., 2016). With this, one might consider that BBV prevalence might be higher in an IVF population than in the general population, since infected men can take advantage of such procedures to prevent the risk of transmitting the virus to partners and offspring. However, it is possible that BBV-infected couples are still unaware of the benefits of such ART techniques, and as such, have not taken the opportunity it offers them to procreate without the risk of viral transmission to their offspring. It is also possible that they are aware of these benefits but are unable to pay for such services. 

It has been reported that male partners of infertile heterosexual relationships may have extra-marital affairs in their quest to achieve pregnancy (Ombelet *et al*., 2008). As such, they are more likely not to use any physical barrier contraception such as condoms and, as a result, are more prone to contract sexually transmitted infections such as BBVs (Ombelet *et al*., 2008). This is evident in the current study, since more males were infected with BBV than their female counterparts (Table 2). Two male patients tested positive for HBsAg on their second attempt at IVF/ICSI, although they were negative during their first attempt. As per standard protocol, all patients who test negative for the Hepatitis B virus are encouraged to receive the vaccination, which was available at the study site. It seems that these two men did not utilize this option. The female partners of these two men tested negative for HBsAg in both cycles, since they received the vaccine after the first testing. 

The study did not find any difference between the sperm quality of BBV infected males and their uninfected counterparts. This supports data from Zhou *et al*. (2011) who also reported no difference in sperm quality between HBV positive and negative males.

There was no significant difference in pregnancy rates between BBV positive couples and their negative counterparts (36.1% vs. 34.9%, *P* = 0.5000). The slight difference in favor of BBV-positive couples could be due to their slightly younger age as compared to their BBV-negative counterparts although again there was no significant difference in their ages (35.9 ± 1.0 vs. 38.2 ± 0.5, *P* = 0.0564 respectively, Table 1).

We hypothesize that due to the relative high cost of IVF procedures, there is the tendency that those who have no children (primary infertility) will have a greater burden to seek ART services than those with secondary infertility (Table 1). 

## CONCLUSION

Access to ARTs is gradually increasing, with about 15 fertility centers in Ghana, although all centers are in the private sector. This current study shows similar BBV prevalence rates in an IVF population and the general population. This underscores the importance of a strict adherence to pre-treatment testing for such viruses, to ensure the safety of personnel and gametes of uninfected patients in these fertility centers. On the basis of viral infectivity, there was no known effect on semen quality. It is, however, noteworthy that these participants were on HAART and these medications could have minimized any adverse effect on semen quality in HIV infected males. A broader study is required to assess semen quality of HIV infected males with and without HAART.
